# Estimating hepatitis B virus prevalence among key population groups for European Union and European Economic Area countries and the United Kingdom: a modelling study

**DOI:** 10.1186/s12879-023-08433-3

**Published:** 2023-07-10

**Authors:** Adam Trickey, Sandra Bivegete, Erika Duffell, Anna L. McNaughton, Lina Nerlander, Josephine G. Walker, Hannah Fraser, Matthew Hickman, Peter Vickerman, Ellen Brooks-Pollock, Hannah Christensen

**Affiliations:** 1 Population Health Sciences, Bristol Medical School, University of Bristol, Bristol, UK; 2 Health Protection Research Unit (HPRU) in Behavioural Science and Evaluation, Bristol, UK; 3 European Centre for Disease Control and Prevention (ECDC), Stockholm, Sweden

**Keywords:** Hepatitis B, Prevalence, Risk groups, General population, Europe, Migrant

## Abstract

**Background:**

Hepatitis B virus (HBV) epidemiology in Europe differs by region and population risk group, and data are often incomplete. We estimated chronic HBV prevalence as measured by surface antigen (HBsAg) among general and key population groups for each country in the European Union, European Economic Area and the United Kingdom (EU/EEA/UK), including where data are currently unavailable.

**Methods:**

We combined data from a 2018 systematic review (updated in 2021), data gathered directly by the European Centre for Disease Control (ECDC) from EU/EEA countries and the UK and further country-level data. We included data on adults from the general population, pregnant women, first time blood donors (FTBD), men who have sex with men (MSM), prisoners, people who inject drugs (PWID), and migrants from 2001 to 2021, with three exceptions made for pre-2001 estimates. Finite Mixture Models (FMM) and Beta regression were used to predict country and population group HBsAg prevalence. A separate multiplier method was used to estimate HBsAg prevalence among the migrant populations within each country, due to biases in the data available.

**Results:**

There were 595 included studies from 31 countries (N = 41,955,969 people): 66 were among the general population (mean prevalence ($\stackrel{-}{p}$) 1.3% [range: 0.0-7.6%]), 52 among pregnant women ($\stackrel{-}{p}=$1.1% [0.1–5.3%]), 315 among FTBD ($\stackrel{-}{p}=$0.3% [0.0-6.2%]), 20 among MSM ($\stackrel{-}{p}=$1.7% [0.0-11.2%]), 34 among PWID ($\stackrel{-}{p}=$3.9% [0.0-16.9%]), 24 among prisoners ($\stackrel{-}{p}=$2.9% [0.0-10.7%]), and 84 among migrants ($\stackrel{-}{p}=$7.0% [0.2–37.3%]). The FMM grouped countries into 3 classes. We estimated HBsAg prevalence among the general population to be < 1% in 24/31 countries, although it was higher in 7 Eastern/Southern European countries. HBsAg prevalence among each population group was higher in most Eastern/Southern European than Western/Northern European countries, whilst prevalence among PWID and prisoners was estimated at > 1% for most countries. Portugal had the highest estimated prevalence of HBsAg among migrants (5.0%), with the other highest prevalences mostly seen in Southern Europe.

**Conclusions:**

We estimated HBV prevalence for each population group within each EU/EAA country and the UK, with general population HBV prevalence to be < 1% in most countries. Further evidence is required on the HBsAg prevalence of high-risk populations for future evidence synthesis.

**Supplementary Information:**

The online version contains supplementary material available at 10.1186/s12879-023-08433-3.

## Introduction

Hepatitis B virus (HBV) is a bloodborne virus that is an important global public health problem, with infections mostly acquired through transmission at birth, injecting drug use, sexual contact, or contaminated blood products and unsafe medical practices [1]. The World Health Organization (WHO) estimating that in 2019, 296 million people had chronic hepatitis B infection [1]. HBV infection can result in liver cirrhosis and hepatocellular carcinoma and is associated with considerable morbidity and mortality, with an estimated 820,000 people globally dying from HBV-related conditions in 2019[1]. The WHO has set global targets for the elimination of hepatitis defined as a 90% reduction in new cases of viral hepatitis and a 65% reduction in deaths from viral hepatitis by 2030, compared with rates in 2015[2]. Other WHO elimination targets are set around HBV vaccination coverage and other prevention measures, diagnosis, and treatment. Based on data collected by the European Centre for Disease Prevention and Control (ECDC), many countries in the European Union (EU)/European Economic Area (EEA) are not currently likely to meet these WHO targets, particularly regarding HBV diagnosis rates [3].

In the EU/EEA and the United Kingdom (UK), the epidemiology of HBV varies between countries, with prevalence among the general population tending to be lower in Western and Northern Europe, and higher in Southern and Eastern Europe [4]. Across the EU/EEA, the prevalence and incidence of HBV is higher in groups such as prisoners, men who have sex with men (MSM), and people who inject drugs (PWID), than in the general population [5, 6]. This variation may be partially explained by differing risk factors, transmission routes, and control strategies across countries. Additionally, migration from high HBV prevalence countries both outside and inside of Europe means that prevalence among migrant populations of some Northern European countries is higher than the corresponding indigenous populations [7].

Due to the varied and dynamic epidemiology of HBV across different countries and population groups in Europe, there is a need for robust estimates of HBV prevalence to aid understanding and support interventions to reduce viral hepatitis to meet the WHO’s 2030 elimination targets [2]. However, data or modelled estimates for each population group of interest are unavailable for many countries [5, 6], particularly for the high-risk populations. In this study we aimed to use statistical modelling to estimate HBsAg prevalence among key population groups for each country in the EU/EEA, as well as the UK, including for countries where empirical data are currently unavailable.

## Methods

This is a country-level ecological study using finite mixture modelling (FMM) to estimate the prevalence of HBsAg among key population groups for countries in the EU/EEA. We collected published and unpublished data on HBsAg prevalence for key population groups for countries in the EU/EEA and the UK. Using a finite mixture modelling (FMM) approach, we produced estimates of HBsAg prevalence for 2019 for key population groups for each country in the EU/EEA and the UK, including those with no data available for a particular population subgroup. A multiplier approach was used to estimate HBsAg prevalence for migrants as the studies available for migrants were deemed unrepresentative of the migrant population in their respective countries, as many of the studies estimated prevalence among specific migrant groups such as those at detention centres.

### Data sources

We included data from the general population and known high-risk populations, encompassing prisoners, PWID, MSM, and migrants [8]. Estimates of prevalence among first time blood donors (FTBD) and pregnant women were included and considered to represent a low-risk population. Whilst the possible bias of estimates among such populations was recognised, it was felt relevant to include these estimates as sources due to their widespread availability. The systematic review and meta-analysis by Bivegete et al. gives further details on which studies were included and their characteristics [11].

Our analysis combined study-level data from several sources (615 studies/estimates in total):

a) A systematic review by the ECDC published in 2016 that had data on HBsAg prevalence for studies among all the groups of interest except for PWID, between 2005 and 2015. The risk of bias was assessed for all these studies. The methods for this search and assessment of risk of bias have been described previously [9]. This systematic review was updated in 2018 to cover studies published 2016–2017, using the same search strategy [10].The systematic review was further updated in 2021 using the same search strategy to include papers published between 2018 and 2021 and the risk of bias was assessed as before [11].

b) Data on prevalence among key population groups gathered through ECDC EU/EEA country contacts. Contacts were sent a summary table containing information already collated from peer-reviewed studies published between 2005 and 2021 and a short questionnaire asking if additional estimates of prevalence were available and what the key drivers for HBV infection were in the country. Out of 30 countries that were contacted, 21 countries responded and shared these additional data sources that were not found through the updated search. To enquire about access to these data, please contact the corresponding author or co-authors from the ECDC.

c) Data on estimates of prevalence of HBV infection among PWID from 2013 to 2019 were obtained from the European Monitoring Centre for Drugs and Drug Addiction (EMCDDA). These estimates are reported to EMCDDA by contact points in EU Member States and Norway. The data were based on observational studies or from routine diagnostic tests offered in drug treatment centres of low-threshold services [12].

For each study we extracted the following variables for use in the statistical models: chronic HBV prevalence estimate (as defined by HBsAg positivity), population group of interest, median/mean age of the individuals included in the study, and the last year of the data collection. For studies included from the systematic reviews we also extracted the risk of bias scores. All additional data from ECDC EU/EEA country contacts were assessed for quality. Further details on determining the risk of bias for each study are given in Bivegete et al [11]. In brief, a previously developed quality assessment framework was adapted [9]. The framework identified key sources of bias in the different risk groups. A scoring system was developed to assess risk of bias with higher scores suggesting lower risk of bias. For the general population, a score between 0 and 6 was used. For studies on pregnant women, we used a score between 0 and 3, whilst this was between 0 and 2 for MSM, and 0 and 6 for people in prison. For estimates included outside the systematic reviews including the data from EMCDDA on PWID and any unpublished data obtained from the country contact data gathering exercise, or for the data among FTBD, the risk of bias was considered to be high as those from the country contacts were mostly unpublished, whilst FTBD are known to be unrepresentative of the general population.

In the statistical model, study-level data were supplemented with the following country-level data to create groups of countries for the model (described in the FMM section below):

• Annual HBV (3-dose) vaccination coverage for 1-year olds for the year 2000 onwards, available from the World Health Organization [13]. Data were missing for some years for some countries, so was categorised as ‘missing’, ‘<90%’, ‘≥90%’ to avoid dropping observations from the model due to missing data.

• The percentage of the local population who are migrants from high HBV endemicity countries (≥ 2% prevalence), taken from a 2016 ECDC report [14].

• Gross domestic product (GDP) per capita in 2019, taken from the World Bank, was included as a marker of socioeconomic development of different countries [15].

• Median age of the country’s population in 2020, taken from the World Factbook [16].

• Latitude and longitude of the country’s midpoint, to capture spatial correlation between countries (https://developers.google.com/public-data/docs/canonical/countries_csv).

### Data processing

There were 19 studies with no data available on the number of participants tested for HBV (but did report prevalence). There were 28 studies that had an estimated HBsAg prevalence of 0%; we assigned a very low prevalence of 0.0001% to these to enable the models to converge. For two unpublished studies where the study year was unknown, we set the year to 2019, to avoid them being dropped from the modelling analyses; 2019 was the year which we used our models to predict the HBsAg prevalence for.

After reviewing the studies among migrant populations, the study team assessed these to overall be unrepresentative of the migrant populations of the studied country, as they included a single group of migrants, or included migrants in reception centres, or recruited from healthcare clinics so were considered a biased sample. These studies were removed from the principal model, although were included in a sensitivity analysis. A separate method for estimating the HBsAg prevalence among the migrant populations of each country is described below.

### Finite mixture modelling

We used FMMs [17] to separate the countries into distinct classes or groups using the country-level variables and then to predict, within each class, the HBsAg prevalence using Beta regression on the study-level variables, which included population group of interest. Beta regression was chosen as we were modelling proportions. This FMM approach allowed us to model unobserved heterogeneity in the data whilst classifying observations and adjusting for clustering. Data for every population group were entered together into the FMMs. This enabled estimates to be produced for each population group of interest in each country and the estimates for one population group of interest to influence those of other groups.

Our FMMs incorporated two steps, the first where the countries were split into separate classes using multivariable models including a spatial component, and the second where estimates were produced for HBsAg prevalence within each separate class, also using multivariable models. For the first stage, we varied the country-level variables (GDP, percentage of the population born in foreign HBV endemic countries, latitude and longitude, age [all continuous], and vaccination coverage category) in the regression models and the number of classes specified (either 3 or 4) to separate the countries into groups. This stage produces distinct groups of countries that are considered similar based on the variables included in the regression model. We also modified the variables included in the second step of regression models that estimated the HBsAg prevalence within each of these classes of countries. These variables were the study-level variables (year of last data collection, population group (excluding migrants), and vaccination coverage category), as well as the latitude and longitude for the centre of each country, and the percentage of the local population who are migrants from high HBV endemicity countries. Model selection was judged by the Akaike Information Criterion (AIC)[18].

For the FMMs, the outcome, HBsAg prevalence, was square-root transformed so that it was approximately normally distributed to aid model convergence. The square-root transformed estimates produced by the FMMs were then untransformed (squared) to calculate country-level HBsAg prevalence estimates. We had initially intended to include study-level data on median/mean age, however, the data were unavailable for 83% of studies, so we excluded this variable.

The FMMs did not explicitly account for country, so all estimates from all countries in each class are used to determine the coefficients for estimating the HBV prevalence within that class. Further details about model selection are given in the supplement. The chosen model was used to predict the HBsAg prevalence for each population subgroup in 2019 for each country using that country’s characteristics for 2019. For each study, we produced a modelled estimate using the study characteristics to compare model fit between the input studies and the modelled output. We compared the general population HBsAg prevalence estimates produced by the FMMs with the corresponding general population estimates from the Institute for Health Metrics and Evaluation (IHME)[19], the Polaris observatory collaborators [20], and Schweitzer et al [21].

### Estimating HBsAg prevalence among migrant populations

As indicated above, we used a separate multiplier method to estimate HBsAg prevalence among the migrant populations within each country (defined as those born outside of their current country of residence), as the studies among migrant populations were deemed overall to be unrepresentative of the migrant populations of the studied country. Similar assumptions have been made by previous studies of HBV prevalence among migrants [22]. This multiplier method did not use data from the studies on migrant populations captured in the search. Information on the percentage of the migrant population by country of birth were retrieved from the European Statistical Office (Eurostat) in each country for 2019[23]. This information was missing for seven countries for which the European Statistical System database was used to retrieve the data of migrant populations by country of birth [24]. Country of origin HBsAg prevalence were extracted from a study of global prevalence conducted by the Polaris observatory collaborators [20]. When prevalence estimates were not reported for some countries, the 2019 modelled estimates were extracted from the IHME [19]. For each country, we multiplied the proportion of the migrant population born in each foreign country with the prevalence in their country of origin and then summed the results to give the estimated prevalence of the overall migrant population in each country.

### Sensitivity analyses

We performed a sensitivity analysis where the studies on migrant populations found in the search were included in the FMM to produce estimates for comparison with the multiplication method for migrants and the main analysis results for other population groups. We intended to use the same FMM produced by the process described above for the main analysis. However, the model selected for the main FMM analysis did not converge, so it was re-run without the median age of the country’s population in 2020 included as a variable to stratify the classes. We had intended to perform a sensitivity analysis only including the studies graded as having a low risk of bias, however, there were too few data points (N = 87), and the models were unable to converge.

Analyses were carried out in Stata version 16.1 (StataCorp, College Station, Texas 77,845 USA).

## Results

### Studies included

There were 432 studies/estimates included from the 2016 and 2018 ECDC reviews, and 41 estimates included from the updated 2021 review. A total of 108 studies were retrieved from ECDC country contacts. Studies from country contacts included mostly FTBD [N = 61], grey literature and unpublished estimates [N = 47]. There were 34 EMCDDA estimates on HBV prevalence among PWID included. Of the potential 615 studies identified from the above sources combined, we excluded 6 studies with missing information on HBsAg prevalence, and 11 further studies from the original review that were only among children aged < 18 (studies that contained both children and adults were included). An additional three duplicate studies were also excluded, leaving 595 studies.

### Characteristics

Of the 595 studies that were included for 30 EU/EEA countries and the UK, 66 studies were on the general population, 52 on pregnant women, 315 on FTBD, 20 on MSM, 34 on PWID, 24 on prisoners, and 84 on migrants (Table 1). Only 11/31 countries had data on MSM, whilst all countries except Liechtenstein had data on FTBD. 21 countries had studies on the general population, 15 on pregnant women, 13 on migrants, 17 on PWID, and 16 among prisoners. The years of the studies ranged from 1987 to 2021. Italy had the most studies (57; 9.6% of total), followed by France and the Netherlands (46; 7.7% each), and Spain (40; 6.7%). Liechtenstein was the only country that had no studies included. Of the 595 estimates included, 156 (26%) were from 2015 or later.

**Table 1:** Number of studies and mean prevalence and range of HBsAg prevalence for each population group of interest, by country (EU/EEA and the UK)

	General population	Pregnant women	First time blood donors	MSM	PWID	Prisoners	Migrants
	N studies: Mean HBsAg prevalence (Range of HBsAg prevalence)						
Austria	0: NA (NA)	0: NA (NA)	7: 0.09% (0.03–0.12)	0: NA (NA)	1: 0.71% (0.71–0.71)	0: NA (NA)	0: NA (NA)
Belgium	2: 0.72% (0.71–0.74)	0: NA (NA)	19: 0.07% (0.04–0.10)	1: 2.30% (2.30–2.30)	1: 1.88% (1.88–1.88)	1: 1.10% (1.10–1.10)	2: 4.62% (2.41–6.83)
Bulgaria	1: 3.93% (3.93–3.93)	1: 2.26% (2.26–2.26)	5: 3.98% (1.83–6.15)	0: NA (NA)	2: 7.26% (5.88–8.64)	0: NA (NA)	0: NA (NA)
Croatia	2: 1.53% (0.75–2.32)	0: NA (NA)	11: 0.14% (0.06–0.23)	2: 2.00% (0.56–3.45)	0: NA (NA)	1: 1.28% (1.28–1.28)	0: NA (NA)
Cyprus	0: NA (NA)	0: NA (NA)	4: 0.24% (0.08–0.44)	0: NA (NA)	2: 9.00% (1.08–16.92)	0: NA (NA)	0: NA (NA)
Czechia	1: 0.34% (0.34–0.34)	0: NA (NA)	11: 0.05% (0.04–0.07)	0: NA (NA)	0: NA (NA)	0: NA (NA)	0: NA (NA)
Denmark	1: 0.24% (0.24–0.24)	2: 0.26% (0.26–0.26)	10: 0.03% (0.00-0.06)	1: 1.42% (1.42–1.42)	0: NA (NA)	0: NA (NA)	1: 6.52% (6.52–6.52)
Estonia	1: 0.00%* (0.00–0.00)*	0: NA (NA)	9: 0.15% (0.07–0.28)	3: 1.47% (0.00-3.38)	2: 6.88% (5.71–8.04)	0: NA (NA)	0: NA (NA)
Finland	0: NA (NA)	1: 0.21% (0.21–0.21)	16: 0.02% (0.00-0.04)	0: NA (NA)	0: NA (NA)	1: 0.52% (0.52–0.52)	1: 1.44% (1.44–1.44)
France	4: 0.97% (0.26–2.16)	3: 0.61% (0.18–0.84)	26: 0.07% (0.03–0.10)	3: 0.76% (0.24–1.37)	0: NA (NA)	3: 1.04% (0.58–1.92)	7: 5.46% (1.93–8.39)
Germany	3: 0.50% (0.30–0.66)	2: 0.64% (0.48–0.80)	11: 0.12% (0.08–0.15)	0: NA (NA)	5: 0.90% (0.40–1.40)	0: NA (NA)	8: 7.42% (2.27–37.25)
Greece	4: 3.95% (1.70–7.55)	6: 2.79% (0.05–5.30)	11: 1.35% (0.45–3.04)	0: NA (NA)	4: 3.48% (1.87–5.26)	1: 8.26% (8.26–8.26)	5: 6.47% (3.16–11.71)
Hungary	1: 0.38% (0.38–0.38)	0: NA (NA)	11: 0.09% (0.00-0.34)	0: NA (NA)	2: 2.21% (2.18–2.24)	1: 1.47% (1.47–1.47)	0: NA (NA)
Iceland	1: 0.17% (0.17–0.17)	0: NA (NA)	10: 0.03% (0.00-0.08)	1: 0.8% (0.80–0.80)	0: NA (NA)	0: NA (NA)	0: NA (NA)
Ireland	3: 0.21% (0.07–0.45)	2: 0.26% (0.21–0.30)	14: 0.02% (0.00-0.04)	0: NA (NA)	0: NA (NA)	1: 0.26% (0.26–0.26)	0: NA (NA)
Italy	13: 1.80% (0.55–5.80)	6: 1.83% (0.47–5.13)	11: 0.21% (0.14–0.33)	1: 11.18% (11.18–11.18)	0: NA (NA)	3: 4.32% (1.86–6.68)	23: 10.18% (2.49–22.73)
Latvia	0: NA (NA)	0: NA (NA)	3: 0.39% (0.38–0.43)	1: 2.00% (2.00–2.00)	2: 2.00% (0.37–3.63)	1: 6.00% (6.00–6.00)	0: NA (NA)
Liechtenstein	0: NA (NA)	0: NA (NA)	0: NA (NA)	0: NA (NA)	0: NA (NA)	0: NA (NA)	0: NA (NA)
Lithuania	0: NA (NA)	0: NA (NA)	7: 0.59% (0.31–0.85)	0: NA (NA)	2: 7.70% (4.90–10.50)	0: NA (NA)	0: NA (NA)
Luxembourg	0: NA (NA)	0: NA (NA)	8: 0.07% (0.00-0.27)	0: NA (NA)	0: NA (NA)	1: 6.96% (6.96–6.96)	0: NA (NA)
Malta	0: NA (NA)	0: NA (NA)	7: 0.19% (0.00-0.46)	0: NA (NA)	0: NA (NA)	0: NA (NA)	1: 6.20% (6.20–6.20)
Netherlands	4: 0.40% (0.22–0.70)	15: 0.31% (0.25–0.38)	15: 0.04% (0.01–0.09)	3: 0.61% (0.48–0.83)	0: NA (NA)	0: NA (NA)	9: 2.99% (0.78–8.46)
Norway	0: NA (NA)	1: 0.06% (0.06–0.06)	11: 0.03% (0.00-0.04)	0: NA (NA)	1: 0.75% (0.75–0.75)	0: NA (NA)	2: 0.91% (0.49–1.34)
Poland	4: 0.99% (0.78–1.12)	1: 0.87% (0.87–0.87)	7: 0.34% (0.17–0.50)	0: NA (NA)	4: 3.34% (2.00-5.41)	1: 3.46% (3.46–3.46)	0: NA (NA)
Portugal	2: 0.90% (0.56–1.23)	0: NA (NA)	5: 0.10% (0.09–0.13)	0: NA (NA)	1: 6.94% (6.94–6.94)	1: 0.66% (0.66–0.66)	0: NA (NA)
Romania	2: 5.01% (4.39–5.64)	1: 5.08% (5.08–5.08)	6: 2.85% (1.84–4.29)	0: NA (NA)	1: 8.78% (8.78–8.78)	1: 10.66% (10.66–10.66)	0: NA (NA)
Slovakia	3: 1.14% (0.10–2.73)	2: 2.23% (2.12–2.34)	11: 0.10% (0.05–0.19)	0: NA (NA)	2: 0.85% (0.00-1.69)	0: NA (NA)	0: NA (NA)
Slovenia	0: NA (NA)	0: NA (NA)	17: 0.07% (0.03–0.11)	0: NA (NA)	0: NA (NA)	0: NA (NA)	0: NA (NA)
Spain	8: 0.53% (0.00-0.88)	5: 0.49% (0.13–0.85)	10: 0.15% (0.12–0.18)	1: 1.55% (1.55–1.55)	1: 7.87% (7.87–7.87)	4: 2.35% (2.10–2.60)	11: 11.91% (2.65–31.74)
Sweden	2: 0.13% (0.06–0.20)	0: NA (NA)	12: 0.04% (0.02–0.06)	0: NA (NA)	1: 2.09% (2.09–2.09)	1: 1.94% (1.94–1.94)	1: 1.59% (1.59–1.59)
UK	4: 0.83% (0.06–1.71)	4: 0.94% (0.29–1.42)	10: 0.03% (0.03–0.04)	3: 0.45% (0.00-1.04)	0: NA (NA)	2: 0.98% (0.00-1.96)	13: 2.96% (0.15–8.73)
**Total**	**66: 1.26% (0.00-7.55)**	**52: 1.07% (0.05–5.30)**	**315: 0.26% (0.00-6.15)**	**20: 1.66% (0.00-11.18)**	**34: 3.90% (0.00-16.92)**	**24: 2.92% (0.00-10.66)**	**84: 6.99% (0.15–37.25)**

*0.00% can be interpreted as < 0.01%

MSM, men who have sex with men; PWID, people who inject drugs

Combining the studies where data were available on number of participants tested, a total of 41,955,969 individuals were included: 2,464,468 (5.9%) as part of general population studies; 3,339,690 (8.0%) pregnant women, 35,465,685 (84.5%) participants in studies among FTBD; 48,582 (0.1%) participants in studies among MSM; 11,785 (0.03%) in studies among PWID; 24,005 (0.06%) in studies among prisoners; and 601,754 (1.4%) in studies among migrants. The mean prevalence reported in the studies in the general population was 1.26% (Range: 0.00-7.55%), 1.07% (0.05–5.30%) among pregnant women, 0.26% (0.00-6.15%) among FTBD, 1.66% (0.00-16.92%) among MSM, 3.90% (0.00-16.92%) among PWID, 2.92% (0.00-10.66%) among prisoners, and 6.99% (0.15–37.25%) among migrants. The prevalence in the included studies ranged from 0 to 37.25%, with most of the studies with the highest prevalence being amongst specific migrant populations. The risk of bias by population group is shown in supplementary Tables 1, with just 10% of studies considered to be low risk of bias.

### Finite mixture modelling

**Fig. 1:**
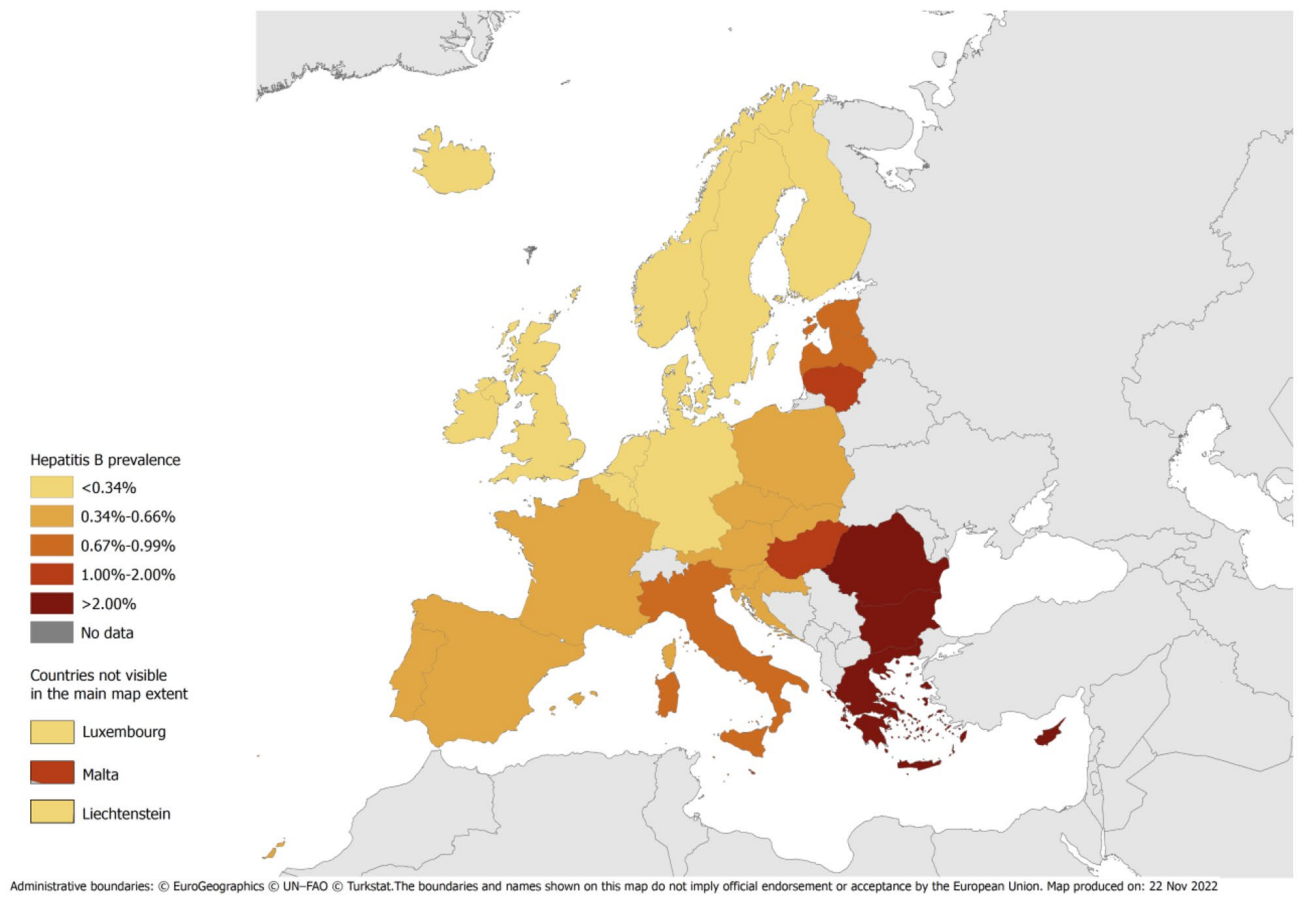
Estimated HBsAg prevalence among the general population

**Fig. 2:**
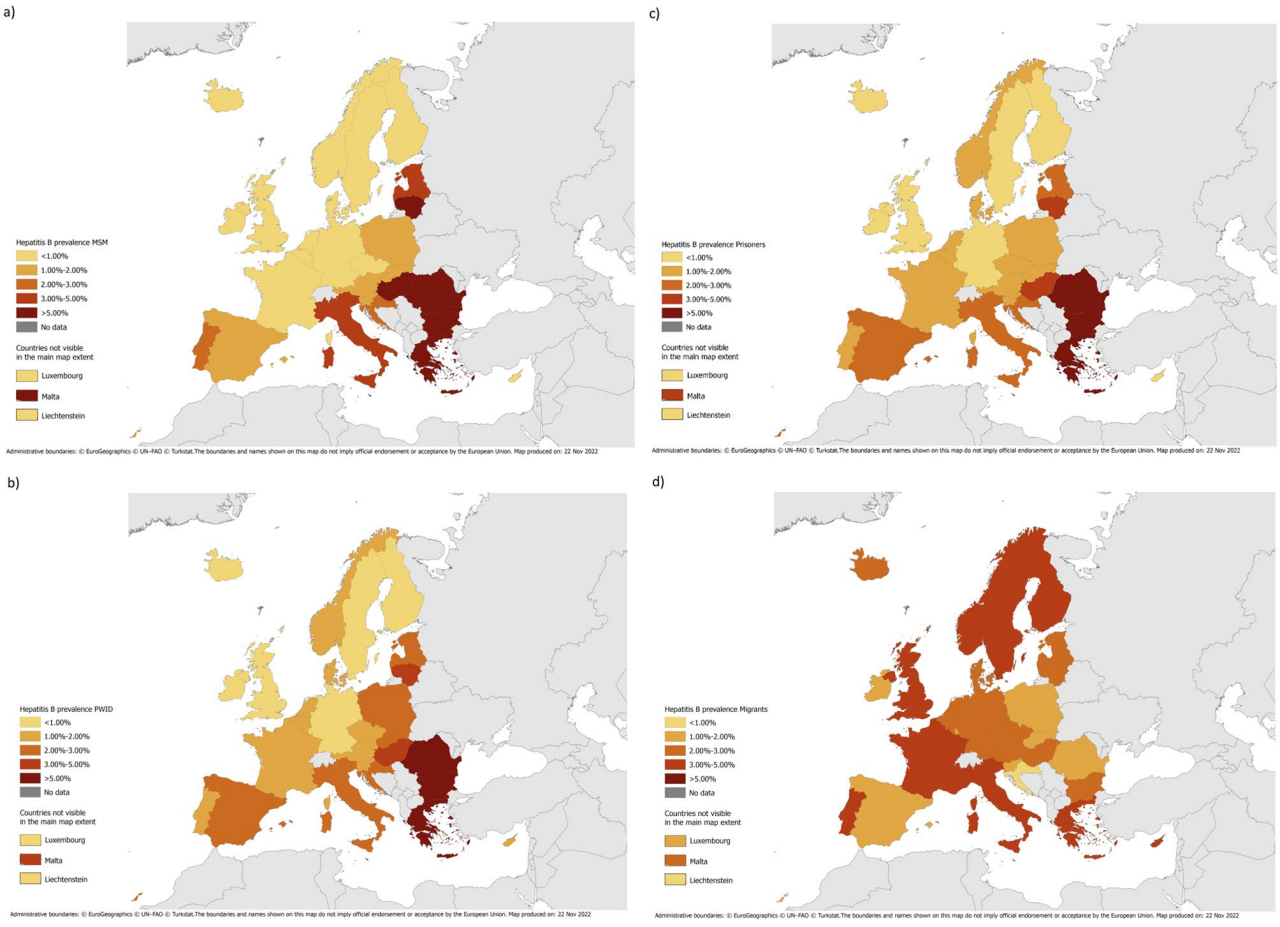
Estimated HBsAg prevalence among **a**) men who have sex with men; **b**) people who inject drugs; **c**) prisoners; and **d**) migrant populations

Table 2; Fig. 1, and Fig. 2a-c presents the results for each population group from the main FMM (that excluded the studies on migrants), with the model coefficients given in supplementary Tables 2 and 3. The chosen model used GDP, percentage of the population born in foreign high HBV endemicity countries, latitude, longitude, and country-level age to separate countries into 3 classes. This chosen model contained study year, population group, vaccination coverage category, latitude, longitude, and the percentage of the population that were migrants from a high HBV endemicity country for the prevalence estimation in each of these 3 classes. Countries in class 1 were Cyprus, Iceland, Ireland, Luxembourg, and Sweden, whilst those in class 2 were mostly from Northern and Western Europe with high GDPs, and the countries in class 3 were mostly from Eastern and Southern Europe.

**Table 2:** Predicted HBsAg prevalence (95% confidence intervals) in 2019 for each population group of interest for each country using the main finite mixture model excluding the migrant studies, and using the multiplication method for the migrant population groups

Class	Country	General population	Pregnant women	FTBD	MSM	PWID	Prisoners	Migrants#
1	Cyprus	6.68% (5.76%, 7.60%)	6.71% (5.90%, 7.53%)	0.35% (0.31%, 0.39%)	0.01% (0.00%, 0.01%)	1.13% (1.06%, 1.20%)	0.01% (0.00%, 0.02%)	3.60%
1	Iceland	0.09% (0.05%, 0.12%)	0.09% (0.06%, 0.11%)	0.00% (0.00%, 0.00%)	0.00% (0.00%, 0.00%)	0.01% (0.01%, 0.01%)	0.00% (0.00%, 0.00%)	2.80%
1	Ireland	0.13% (0.10%, 0.17%)	0.14% (0.11%, 0.16%)	0.00% (0.00%, 0.01%)	0.00% (0.00%, 0.00%)	0.02% (0.01%, 0.02%)	0.00% (0.00%, 0.00%)	1.82%
1	Luxembourg	0.25% (0.20%, 0.31%)	0.26% (0.22%, 0.29%)	0.01% (0.01%, 0.01%)	0.00% (0.00%, 0.00%)	0.03% (0.03%, 0.04%)	0.00% (0.00%, 0.00%)	1.86%
1	Sweden	0.15% (0.11%, 0.20%)	0.15% (0.12%, 0.19%)	0.01% (0.00%, 0.01%)	0.00% (0.00%, 0.00%)	0.02% (0.02%, 0.02%)	0.00% (0.00%, 0.00%)	3.50%
2	Belgium	0.33% (0.32%, 0.33%)	0.38% (0.38%, 0.38%)	0.05% (0.05%, 0.05%)	0.80% (0.79%, 0.80%)	1.59% (1.58%, 1.61%)	1.35% (1.34%, 1.36%)	2.80%
2	Czechia	0.39% (0.38%, 0.39%)	0.45% (0.44%, 0.45%)	0.06% (0.06%, 0.06%)	0.94% (0.93%, 0.95%)	1.86% (1.84%, 1.88%)	1.58% (1.56%, 1.59%)	2.93%
2	Denmark	0.25% (0.25%, 0.25%)	0.29% (0.29%, 0.30%)	0.04% (0.04%, 0.04%)	0.62% (0.61%, 0.63%)	1.25% (1.23%, 1.26%)	1.05% (1.04%, 1.07%)	2.89%
2	France	0.39% (0.39%, 0.39%)	0.45% (0.45%, 0.46%)	0.06% (0.06%, 0.06%)	0.95% (0.94%, 0.95%)	1.88% (1.86%, 1.90%)	1.59% (1.59%, 1.60%)	3.39%
2	Netherlands	0.24% (0.24%, 0.24%)	0.28% (0.28%, 0.28%)	0.03% (0.03%, 0.03%)	0.60% (0.59%, 0.60%)	1.21% (1.20%, 1.22%)	1.02% (1.01%, 1.03%)	2.96%
2	Norway	0.27% (0.27%, 0.28%)	0.32% (0.31%, 0.33%)	0.04% (0.04%, 0.04%)	0.68% (0.66%, 0.69%)	1.36% (1.34%, 1.38%)	1.15% (1.13%, 1.17%)	3.39%
2	Poland	0.42% (0.41%, 0.43%)	0.49% (0.48%, 0.50%)	0.06% (0.06%, 0.06%)	1.02% (1.01%, 1.04%)	2.02% (2.00%, 2.04%)	1.71% (1.69%, 1.74%)	1.92%
2	Slovakia	0.47% (0.46%, 0.48%)	0.54% (0.53%, 0.55%)	0.07% (0.07%, 0.07%)	1.13% (1.11%, 1.15%)	2.22% (2.19%, 2.24%)	1.88% (1.86%, 1.91%)	1.24%
2	Spain	0.51% (0.50%, 0.51%)	0.59% (0.58%, 0.59%)	0.07% (0.07%, 0.07%)	1.22% (1.21%, 1.23%)	2.38% (2.36%, 2.41%)	2.03% (2.02%, 2.04%)	1.96%
2	UK	0.19% (0.19%, 0.19%)	0.22% (0.22%, 0.23%)	0.03% (0.03%, 0.03%)	0.48% (0.48%, 0.48%)	0.97% (0.96%, 0.98%)	0.82% (0.81%, 0.83%)	3.29%
3	Austria	0.34% (0.32%, 0.35%)	0.37% (0.36%, 0.39%)	0.09% (0.09%, 0.10%)	1.59% (1.54%, 1.65%)	1.20% (1.18%, 1.22%)	1.18% (1.15%, 1.22%)	2.15%
3	Bulgaria	2.04% (1.92%, 2.16%)	2.24% (2.16%, 2.33%)	0.62% (0.60%, 0.64%)	7.91% (7.62%, 8.21%)	6.23% (6.12%, 6.34%)	6.16% (5.96%, 6.36%)	2.24%
3	Croatia	0.61% (0.59%, 0.63%)	0.68% (0.66%, 0.70%)	0.17% (0.17%, 0.18%)	2.77% (2.69%, 2.84%)	2.10% (2.07%, 2.13%)	2.08% (2.03%, 2.12%)	0.76%
3	Estonia	0.82% (0.78%, 0.86%)	0.90% (0.87%, 0.94%)	0.23% (0.23%, 0.24%)	3.58% (3.49%, 3.68%)	2.74% (2.69%, 2.79%)	2.71% (2.63%, 2.79%)	2.13%
3	Finland	0.16% (0.12%, 0.20%)	0.18% (0.14%, 0.21%)	0.04% (0.04%, 0.05%)	0.79% (0.66%, 0.91%)	0.58% (0.50%, 0.67%)	0.58% (0.48%, 0.68%)	3.73%
3	Germany	0.20% (0.19%, 0.21%)	0.22% (0.21%, 0.23%)	0.05% (0.05%, 0.06%)	0.98% (0.95%, 1.02%)	0.73% (0.72%, 0.74%)	0.72% (0.70%, 0.74%)	2.51%
3	Greece	2.26% (2.19%, 2.32%)	2.48% (2.43%, 2.53%)	0.69% (0.68%, 0.70%)	8.60% (8.41%, 8.79%)	6.80% (6.75%, 6.85%)	6.72% (6.61%, 6.84%)	4.11%
3	Hungary	1.38% (1.32%, 1.43%)	1.52% (1.48%, 1.55%)	0.41% (0.40%, 0.41%)	5.65% (5.51%, 5.80%)	4.39% (4.35%, 4.43%)	4.34% (4.26%, 4.43%)	2.97%
3	Italy	0.75% (0.73%, 0.78%)	0.83% (0.81%, 0.85%)	0.22% (0.21%, 0.22%)	3.33% (3.24%, 3.42%)	2.54% (2.51%, 2.57%)	2.51% (2.46%, 2.56%)	4.00%
3	Latvia	0.89% (0.84%, 0.93%)	0.98% (0.95%, 1.01%)	0.26% (0.25%, 0.26%)	3.85% (3.76%, 3.95%)	2.95% (2.91%, 3.00%)	2.92% (2.84%, 3.00%)	2.53%
3	Liechtenstein	0.05% (0.04%, 0.06%)	0.05% (0.04%, 0.06%)	0.01% (0.01%, 0.01%)	0.24% (0.20%, 0.28%)	0.18% (0.15%, 0.20%)	0.18% (0.15%, 0.20%)	0.09%
3	Lithuania	1.41% (1.33%, 1.49%)	1.55% (1.50%, 1.60%)	0.42% (0.40%, 0.43%)	5.76% (5.60%, 5.93%)	4.48% (4.42%, 4.54%)	4.43% (4.32%, 4.54%)	2.54%
3	Malta	1.45% (1.40%, 1.50%)	1.60% (1.56%, 1.64%)	0.43% (0.42%, 0.44%)	5.92% (5.74%, 6.10%)	4.61% (4.55%, 4.66%)	4.55% (4.46%, 4.65%)	2.40%
3	Portugal	0.57% (0.55%, 0.59%)	0.63% (0.61%, 0.65%)	0.16% (0.16%, 0.16%)	2.58% (2.49%, 2.67%)	1.96% (1.92%, 1.99%)	1.93% (1.89%, 1.98%)	5.00%
3	Romania	2.87% (2.74%, 3.01%)	3.15% (3.07%, 3.23%)	0.89% (0.87%, 0.92%)	10.5% (10.23%, 10.77%)	8.38% (8.30%, 8.45%)	8.29% (8.12%, 8.46%)	1.31%
3	Slovenia	0.40% (0.38%, 0.42%)	0.45% (0.43%, 0.46%)	0.11% (0.11%, 0.11%)	1.88% (1.82%, 1.94%)	1.42% (1.39%, 1.44%)	1.40% (1.36%, 1.44%)	1.05%

FTBD: First-time blood donors. MSM: Men who have sex with men. PWID: People who inject drugs

# Confidence intervals are not presented for migrants due to use of the separate multiplication method

Cyprus had a much higher estimated general population HBV prevalence than other countries, 6.68% (5.76-7.60%), with the second highest being Romania with 2.87% (2.74-3.01%). MSM, PWID, and prisoners tended to have higher estimated prevalence than the other groups, although the estimates for countries in class 1 were all < 0.03%, with the exception of PWID in Cyprus. For each population group, countries in class 3 tended to have higher estimates than countries in the other classes and prevalences were mostly estimated to be higher among Eastern and Southern European countries compared to Western and Northern European countries. Prevalences among PWID and prisoners were > 1% for most countries.

**Fig. 3:**
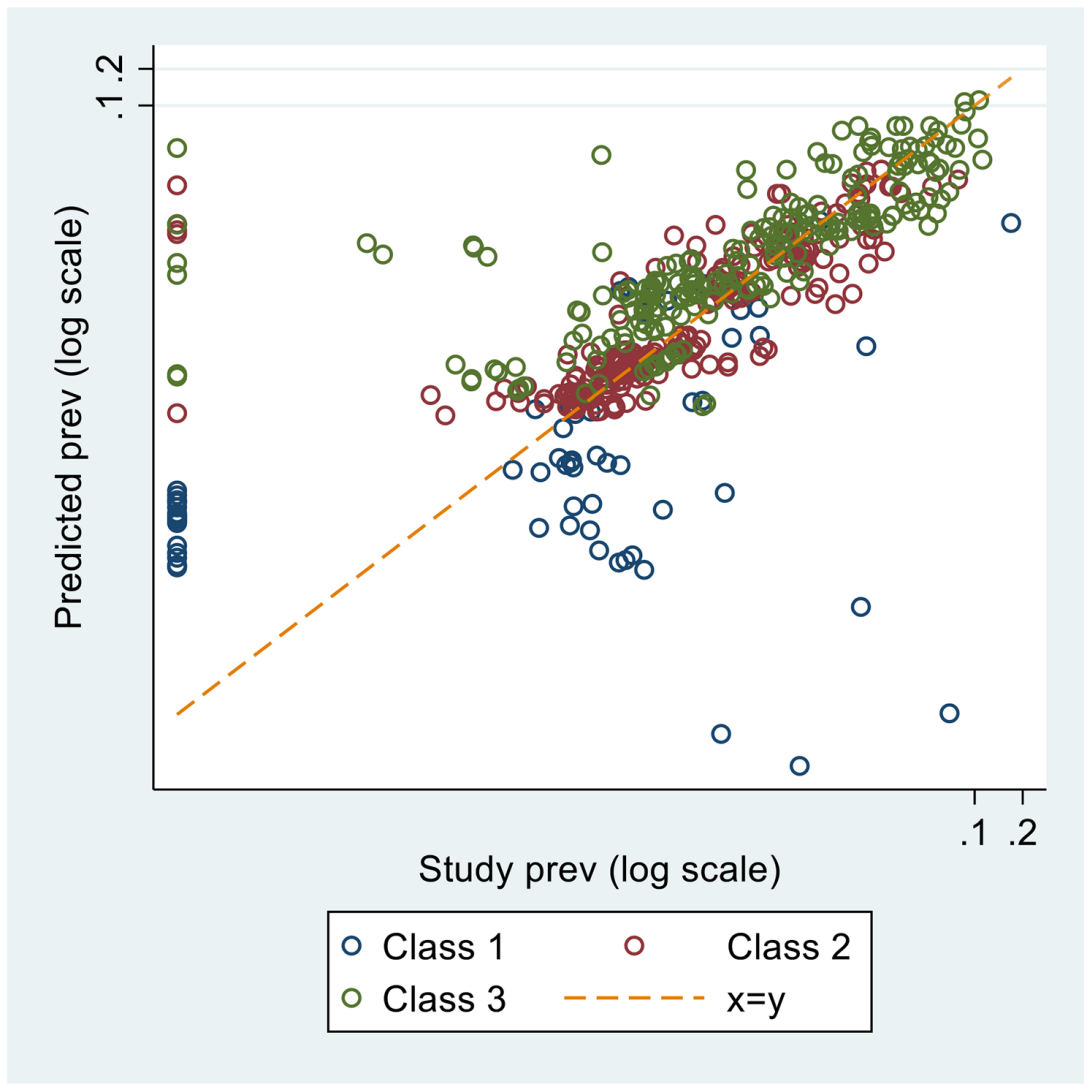
Predicted HBsAg prevalence versus actual study prevalence by model for all population groups, except migrants – on a log scale. Countries in class 1 were Cyprus, Iceland, Ireland, Luxembourg, and Sweden, whilst those in class 2 were mostly from Northern and Western Europe with high GDPs, and the countries in class 3 were mostly from Eastern and Southern Europe

Figure 3 and supplementary Fig. 1 show a comparison of the predicted versus observed HBsAg prevalence at the study-level, overall and split by class; the fitted values were sub-optimal for FTBD in class 1. The median difference between the observed and predicted prevalence was 0.007% (interquartile range: -0.057%, 0.129%). The estimates for FTBD generally had the lowest prevalence (all < 1%), followed by the general population and pregnant women. 24/31 countries had general population prevalences < 1%, with the exceptions occurring in Eastern/Southern Europe.

### Estimates for migrants

The estimated HBsAg prevalence among migrant populations in each country using the multiplication method are shown in Table 2; Fig. 2d, with the highest estimated prevalence being seen in Portugal (5.00%) and the lowest in Liechtenstein (0.09%). The highest estimated HBV prevalences among migrants were for countries in Southern Europe, although some Scandinavian countries also had high estimated prevalences among migrants.

### Comparisons with other general population estimates

Table 3 gives a comparison of the estimated HBsAg prevalence for 2019 among the general population using the FMM, compared with estimates for the general population taken from Schweitzer et al [21], the Polaris Observatory [20], and the Institute for Health Metrics and Evaluation (IHME)[19], although estimates were only available across all countries for our study. Overall IHME estimated the highest prevalences for the most countries, 10/30, whilst our estimates were the lowest for 12/30 countries (excluding Liechtenstein where we were the only study to produce estimates).

**Table 3:** Comparison of the general population HBsAg prevalence data; the estimates (95% confidence intervals) from this research, and those of Schweitzer et al [21], Polaris [20], and the Institute of Health Metrics and Evaluation (IHME)[19]. Bold indicates the highest estimate across the four studies, and italics indicates the lowest estimate

Class	Country	General population data; mean (range) HBsAg prevalence	Present study	Schweitzer et al.	Polaris	IHME
1	Cyprus	Unavailable	**6.68% (5.76%, 7.60%)**	2.69% (2.38–3.04)	Unavailable	*0.70% (0.67–0.72)*
1	Iceland	0.17% (0.17%, 0.17%)	*0.09% (0.05%, 0.12%)*	0.14% (0.04–0.56)	Unavailable	**0.71% (0.58–0.84)**
1	Ireland	0.21% (0.07%, 0.45%)	0.13% (0.10%, 0.17%)	*0.03% (0.01–0.07)*	0.1% (0.1–0.1)	**0.69% (0.55–0.81)**
1	Luxembourg	Unavailable	*0.25% (0.20%, 0.31%)*	Unavailable	Unavailable	**0.82% (0.68–0.96)**
1	Sweden	Unavailable	*0.15% (0.11%, 0.20%)*	**0.59% (0.48–0.73)**	0.2% (0.1–0.2)	0.58% (0.50–0.67)
2	Belgium	0.72% (0.71%, 0.74%)	*0.33% (0.32%, 0.33%)*	**0.68% (0.47–0.99)**	0.6% (0.5–0.7)	0.59% (0.54–0.65)
2	Czechia	0.34% (0.34%, 0.34%)	*0.39% (0.38%, 0.39%)*	**1.24% (0.98–1.56)**	0.4% (0.2–0.5)	0.71% (0.61–0.79)
2	Denmark	0.24% (0.24%, 0.24%)	*0.25% (0.25%, 0.25%)*	**0.91% (0.87–0.95)**	0.3% (0.2–0.3)	0.89% (0.74–1.05)
2	France	0.97% (0.26%, 2.16%)	0.39% (0.39%, 0.39%)	*0.26% (0.25–0.27)*	0.5% (0.4–0.7)	**1.38% (1.30–1.44)**
2	Netherlands	0.40% (0.22%, 0.70%)	*0.24% (0.24%, 0.24%)*	0.40% (0.39–0.41)	0.3% (0.1–0.4)	**0.94% (0.77–1.13)**
2	Norway	Unavailable	0.27% (0.27%, 0.28%)	*0.01% (0.00-0.03)*	0.3% (0.3–0.4)	**0.56% (0.48–0.64)**
2	Poland	0.99% (0.78%, 1.12%)	*0.42% (0.41%, 0.43%)*	*0.42% (0.42–0.43)*	**0.9% (0.7–1.1)**	0.44% (0.39–0.49)
2	Slovakia	1.14% (0.10%, 2.73%)	*0.47% (0.46%, 0.48%)*	**1.74% (1.64–1.85)**	1.6% (0.7–1.8)	0.81% (0.69–0.93)
2	Spain	0.53% (0.00%, 0.88%)	0.51% (0.50%, 0.51%)	*0.34% (0.32–0.37)*	0.6% (0.4–0.9)	**0.82% (0.73–0.91)**
2	United Kingdom	0.83% (0.06%, 1.71%)	0.19% (0.19%, 0.19%)	*0.01% (0.01–0.01)*	0.7% (0.5–0.9)	**0.77% (0.67–0.88)**
3	Austria	Unavailable	*0.34% (0.32%, 0.35%)*	**1.23% (0.81–1.86)**	Unavailable	0.82% (0.66–0.97)
3	Bulgaria	3.93% (3.93%, 3.93%)	*2.04% (1.92%, 2.16%)*	**3.92% (3.19–4.81)**	3.2% (1.9–5.6)	2.59% (2.47–2.69)
3	Croatia	1.53% (0.75%, 2.32%)	0.61% (0.59%, 0.63%)	1.11% (0.95–1.30)	*0.6% (0.5-1.0)*	**1.17% (1.06–1.26)**
3	Estonia	0.00% (0.00%, 0.00%)	**0.82% (0.78%, 0.86%)**	Unavailable	*0.5% (0.5–0.6)*	0.53% (0.45–0.61)
3	Finland	Unavailable	*0.16% (0.12%, 0.20%)*	Unavailable	0.2% (0.2–0.2)	**0.96% (0.81–1.12)**
3	Germany	0.50% (0.30%, 0.66%)	*0.20% (0.19%, 0.21%)*	0.70% (0.65–0.76)	0.3% (0.2–0.6)	0.39% (0.34–0.44)
3	Greece	3.95% (1.70%, 7.55%)	**2.26% (2.19%, 2.32%)**	*0.97% (0.95-1.00)*	1.8% (1.5-2.0)	1.95% (1.78–2.13)
3	Hungary	0.38% (0.38%, 0.38%)	**1.38% (1.32%, 1.43%)**	0.53% (0.46–0.61)	*0.4% (0.4–0.5)*	0.68% (0.63–0.74)
3	Italy	1.80% (0.55%, 5.80%)	0.75% (0.73%, 0.78%)	**2.52% (2.49–2.54)**	*0.6% (0.3–0.7)*	1.00% (NA-NA)
3	Latvia	Unavailable	**0.89% (0.84%, 0.93%)**	Unavailable	Unavailable	*0.54% (0.46–0.61)*
3	Liechtenstein	Unavailable	***0.05% (0.04%, 0.06%)***	Unavailable	Unavailable	Unavailable
3	Lithuania	Unavailable	1.41% (1.33%, 1.49%)	**1.70% (1.55–1.86)**	Unavailable	*0.55% (0.47–0.64)*
3	Malta	Unavailable	**1.45% (1.40%, 1.50%)**	Unavailable	Unavailable	*0.65% (0.54–0.77)*
3	Portugal	0.90% (0.56%, 1.23%)	*0.57% (0.55%, 0.59%)*	1.02% (0.78–1.31)	**1.2% (0.9–1.5)**	0.91% (0.74–1.09)
3	Romania	5.01% (4.39%, 5.64%)	2.87% (2.74%, 3.01%)	**5.61% (5.50–5.73)**	3.4% (3.2–3.7)	*1.05% (0.89–1.19)*
3	Slovenia	Unavailable	0.40% (0.38%, 0.42%)	*0.28% (0.25–0.30)*	**1.0% (0.4–1.1)**	0.92% (0.80–1.03)

### Sensitivity analysis

The results of the sensitivity analysis when including the studies among migrants in the FMM are shown in Supplementary Tables 4, 5 and 6; 22 (71%) of countries were placed in the same class (supplementary Table 6). Some large differences between the sensitivity analysis and the baseline FMM results are seen for each key population group of interest, with the estimates generally being higher in the sensitivity analysis. In particular, the estimated HBsAg prevalence among migrant populations are much higher for many countries using this FMM approach including migrant studies with 11 countries having an estimated prevalence > 10% based on the FMM approach compared to 0 countries based on the multiplication approach.

## Discussion

In this study we use innovative approaches to synthesise and estimate HBsAg prevalence in 2019 for EU/EEA countries and the UK for the general population, low-risk populations (pregnant women and FTBD) and several high-risk groups (MSM, PWID, prisoners, and migrants), including in groups where estimates were previously unavailable. Our study also highlights the current data gaps, with 11/31 countries having data on HBsAg prevalence among MSM, whilst 30 countries had data on FTBD, 21 countries had studies on the general population, 17 on PWID, and 16 among prisoners, 15 on pregnant women, and just 13 on migrants, whilst only 156/595 of the included estimates contained data from 2015 onwards. We estimated the HBsAg prevalence among the general population to be < 1% in 24/31 countries, but it was higher in 7 Eastern and Southern European countries. HBsAg prevalences for all population groups are estimated to be higher in most Eastern and Southern European countries than those in Western and Northern Europe, whilst prevalences among PWID and prisoners tend to be > 1% for most countries. The countries with the highest estimated prevalences of HBsAg among migrants were mostly in Southern Europe, although estimates were also high for some Scandinavian countries.

### Comparison with other literature

Other studies have estimated HBsAg prevalence for multiple countries across the world or Europe for either the general population or high-risk groups. The Polaris Observatory estimated HBV prevalence using a systematic review combined with a compartmental infectious disease modelling approach [20], whilst the IHME also used a modelling approach to estimate HBV prevalence [19], and Schweitzer et al [21] produced estimates using a systematic review and meta-analysis for the WHO Global Hepatitis Report 2017[25]. The estimates produced by these studies differed substantially from each other, particularly for Eastern European countries. They also produced general population estimates that tended to be lower than our modelled estimates, potentially because we synthesised information from other population groups rather than solely using information from the general population. Our estimates for Western European countries were similar to those produced by the Polaris Observatory. High-risk groups, particularly migrant populations, can form a large percentage of a country’s HBV burden, particularly in low prevalence countries [14] even if the overall population burden is low. However, this will have less influence on a country’s HBV burden when the general population prevalence is high and/or the number of migrants from high endemicity countries is low, such as in many countries in Eastern Europe. Previous literature has found that countries in sub-Saharan Africa and East Asia have much higher prevalences of HBV than countries in Europe [21]. Falla et al’s systematic review on the HBsAg prevalence among multiple high-risk groups used similar data but did not synthesise estimates; multiple estimates presented per country make it difficult to compare with our results [6]. A systematic review and meta-analysis of studies among PWID by Degenhardt et al. estimated HBsAg prevalences worldwide and generally found lower prevalences than we estimated for Eastern European countries, but higher prevalences in Western European countries [26]. Dolan et al’s systematic review estimated the HBsAg prevalences among prisoners globallyand found similar prevalences to our results [27]. Ahmad et al. used different methods to estimate the prevalence among migrant populations in each country and tended to find higher prevalences than our estimates for HBV prevalence among migrants [22].

### Strengths and limitations

Strengths of this study include the innovative approach and large sample size. We acknowledge several limitations of our methodological approach. Model fitting was challenging, leading to few of the considered models to converge, which gave less options for model selection. Information for the UK was not captured in the updated search for 2018–2021, which may have created a bias, as no recent studies would have been included. Our multiplication method for countering the unrepresentative nature of the migrant studies may have had limitations, such as not accounting for the healthy migrant effect. However, for this process we did not have information on the age breakdown of migrants, which could have affected these estimates, as HBsAg prevalences are likely higher in older population groups who have not benefited from immunisation. There was no evidence from general population surveys for 10/31 countries and 35/66 general population surveys were from before 2010, whilst 5/20 and 9/24 studies among MSM and prisoners, respectively, were from pre-2010, which may have resulted in overestimates of prevalence, although we did account for study year. Ideally these would be updated or recent data available from screening of pregnant women by ethnic group that could be used to supplement general population surveys.

## Conclusions

This study provides an innovative modelling approach and a complete range of HBV prevalence estimates for the general population and all high-risk key population groups for all EU/EEA countries and the UK. There are crucial gaps in the primary data in many countries, particularly for MSM, migrant populations, PWID, and prisoners, where robust and up-to-date studies on HBsAg prevalence are required. Prevalence estimates for the general population vary substantially between different studies [19–21], underlining the paucity of HBsAg prevalence estimates in this group. Our study is of value to policy makers that need to plan interventions to achieve the WHO’s 2030 goals for the elimination of viral hepatitis [2]. The prevalence of HBV in Europe is likely falling over time due to the effect of HBV birth vaccinations, although this may be affected by migration from countries where HBV is endemic [22]. HBV among non-foreign-born populations is concentrated among key population groups, in which resources for HBV surveillance should be prioritised [6]. Our estimates provide data to policy makers on the scale of the HBV epidemic among the different populations and highlight which groups should be targeted for prevention and control interventions, including testing to identify individuals with HBV who should be linked to care and treated with antivirals where relevant.

## Electronic supplementary material

Below is the link to the electronic supplementary material.

Supplementary Material 1

## Data Availability

All data generated or analysed during this study are included in this published article and its supplementary information files.

## Abbreviations

AIC: Akaike’s Information Criterion
ECDC: European Centre for Disease Prevention and Control
EMCDDA: European Monitoring Centre for Drugs and Drug Addiction
EU: European Union
EEA: European Economic Area
FMM: Finite Mixture Modelling
FTBD: First-time blood donors
GDP: Gross Domestic Product per capita
HBsAg: Hepatitis B surface antigen
HBV: Hepatitis B virus
IHME: Institute for Health Metrics and Evaluation
MSM: Men who have sex with men
PWID: People who inject drugs
UK: United Kingdom
WHO: World Health Organization
