# Supplementary material for: Estimating hepatitis B virus prevalence among key population groups for European Union and European Economic Area countries and the United Kingdom: a modelling study
Source: BMC Infect Dis. 2023 Jul 10;23:457. doi: 10.1186/s12879-023-08433-3 (PMC10331985; doi:10.1186/s12879-023-08433-3)
Supplement: Supplementary file 1 — Supplementary Material 1 [file 12879_2023_8433_MOESM1_ESM.docx]

**Estimating hepatitis B virus prevalence among key population groups for European Union and European Economic Area countries and the United Kingdom: a modelling study**

**Supplementary material**

**Risk of bias**

**Supplementary table 1:** Risk of bias scores by population group.

| **Risk of bias** | **FTBD*** | **General population** | **MSM** | **PWID*** | **Pregnant women** | **Prisoners** | **Total** |
| --- | --- | --- | --- | --- | --- | --- | --- |
| **High** | 315 (100%) | 25 (38%) | 7 (35%) | 34 (100%) | 16 (31%) | 2 (8%) | 399 (78%) |
| **Low** | 0 (0%) | 20 (30%) | 4 (20%) | 0 (0%) | 15 (29%) | 10 (42%) | 49 (10%) |
| **Missing** | 0 (0%) | 21 (32%) | 9 (45%) | 0 (0%) | 21 (40%) | 12 (50%) | 63 (12%) |
| **Total** | 0 (0%) | 66 (100%) | 20 (100%) | 34 (100%) | 52 (100%) | 24 (100%) | 511 (100%) |

FTBD: First time blood donors

MSM: Men who have sex with men

PWID: People who inject drugs

* All studies among FTBD and PWID were considered high-risk.

**Selecting the Finite Mixture Model**

Model fitting was challenging, and few models were able to converge. The models that were able to converge were:

- “Basic vars; 3 class”. Class selection variables: GDP, percentage of the population born in foreign HBV endemic countries, latitude, and longitude (all continuous). Prevalence estimation variables: Study year, population group, vaccination coverage category, latitude, longitude.
- “Age added; 3 class”. Class selection variables as in “Basic vars; 3 class”, but also including country median age. Prevalence estimation variables as in “Basic vars; 3 class”
- “HBV endemic migrant % added; 3 class”. Class selection variables as in “Basic vars; 3 class”. Prevalence estimation variables as in “Basic vars; 3 class” adding the percentage of the population that were migrants from a high HBV endemicity country.
- **“Age and HBV endemic migrant % added; 3 class”. Class selection variables as in “Basic vars; 3 class”, but also including country median age. Prevalence estimation variables as in “Basic vars; 3 class” adding the percentage of the population that were migrants from a high HBV endemicity country. Chosen using Akaike’s Information Criterion.**
- “HBV endemic migrant % added; 4 class”. The same as “HBV endemic migrant % added; 3 class”, but with 4 classes.

The models that were not able to converge were:

- “Vacc added; 3 class” – 3 classes. Class selection variables as in “Basic vars; 3 class”, but also including the vaccination coverage categories. Prevalence estimation variables as in “Basic vars; 3 class”.
- “Vacc and HBV endemic migrant % added; 3 class” – 3 classes. Class selection variables as in “Basic vars; 3 class”, but also including the vaccination coverage categories. Prevalence estimation variables as in “Basic vars; 3 class” adding the percentage of the population that were migrants from a high HBV endemicity country.
- “Age and HBV endemic migrant % added; 3 class” – 3 classes. Class selection variables as in “Basic vars; 3 class”, but also including country median age. Prevalence estimation variables as in “Basic vars; 3 class” adding the percentage of the population that were migrants from a high HBV endemicity country.
- “Age, vacc, and HBV endemic migrant % added; 3 class” – 3 classes. Class selection variables as in “Basic vars; 3 class”, but also including country median age and the vaccination coverage categories. Prevalence estimation variables as in “Basic vars; 3 class” adding the percentage of the population that were migrants from a high HBV endemicity country.
- “Basic vars; 4 class”. The same as “Basic vacs; 3 class” but with 4 classes.
- “Age added; 4 class”. The same as “Age added; 3 class” but with 4 classes.
- “Vacc added; 4 class”. The same as “Vacc added; 3 class” but with 4 classes.
- “Age and HBV endemic migrant % added; 4 class”. The same as “Age and HBV endemic migrant % added; 3 class” but with 4 classes.
- “Vacc and HBV endemic migrant % added; 4 class”. The same as “Vacc and HBV endemic migrant % added; 3 class” but with 4 classes.
- “Age and vacc added; 4 class”. The same as “Age and vacc added; 3 class” but with 4 classes.
- “Age, vacc and HBV endemic migrant % added; 4 class”. The same as “Age, vacc and HBV endemic migrant % added; 3 class” but with 4 classes.

**Fitted model output:**

**Supplementary table 2:** Output selecting studies into classes for model “Age added; 3 class”

|  | **Coef.** | **Std. Err.** | **z** | **P>\|z\|** | **95%LI** | **95%UI** |
| --- | --- | --- | --- | --- | --- | --- |
| **1.Class** | Base outcome | |  |  |  |  |
| **2.Class** |  |  |  |  |  |  |
| gdp2019 | -1.7E-05 | 1.47E-05 | -1.19 | 0.234 | -4.6E-05 | 1.13E-05 |
| migrant_endemic | -0.65403 | 0.164196 | -3.98 | 0 | -0.97584 | -0.33221 |
| Latitude | 0.184329 | 0.053929 | 3.42 | 0.001 | 0.07863 | 0.290027 |
| Longitude | -0.23705 | 0.052544 | -4.51 | 0 | -0.34003 | -0.13407 |
| Median_age | 0.624396 | 0.177821 | 3.51 | 0 | 0.275873 | 0.972919 |
| _cons | -28.0354 | 8.952515 | -3.13 | 0.002 | -45.582 | -10.4888 |
| **3.Class** |  |  |  |  |  |  |
| gdp2019 | 1.93E-05 | 0.000016 | 1.21 | 0.228 | -1.2E-05 | 5.06E-05 |
| migrant_endemic | -0.17674 | 0.087631 | -2.02 | 0.044 | -0.34849 | -0.00499 |
| Latitude | -0.06569 | 0.046751 | -1.41 | 0.16 | -0.15733 | 0.025936 |
| Longitude | 0.085046 | 0.043949 | 1.94 | 0.053 | -0.00109 | 0.171184 |
| Median_agei | 0.832976 | 0.175694 | 4.74 | 0 | 0.488621 | 1.177331 |
| _cons | -32.0052 | 8.058641 | -3.97 | 0 | -47.7998 | -16.2105 |

LI: Lower interval. UI: Upper interval.

**Supplementary table 3:** Study-level output predicting the square-root transformed HBsAg prevalence from the model for a) Class 1; b) Class 2: and c) Class 3.

1. Class 1

| **Variable** | **Coef.** | **Std. Err.** | **z** | **P>\|z\|** | **95%LI** | **95%UI** |
| --- | --- | --- | --- | --- | --- | --- |
|  |  |  |  |  |  |  |
| Year | 0.0230029 | 0.0169633 | 1.36 | 0.175 | -0.0102446 | 0.0562504 |
|  |  |  |  |  |  |  |
| Population group |  |  |  |  |  |  |
| First time blood donors | Comparator |  |  |  |  |  |
| General population | 1.708695 | 0.1927756 | 8.86 | 0 | 1.330862 | 2.086528 |
| MSM | -2.178806 | 0.6852273 | -3.18 | 0.001 | -3.521826 | -0.8357849 |
| PWID | 0.6340178 | 0.2889205 | 2.19 | 0.028 | 0.067744 | 1.200292 |
| Pregnant women | 1.711759 | 0.2709561 | 6.32 | 0 | 1.180695 | 2.242823 |
| Prisoners | -1.929378 | 0.9298253 | -2.07 | 0.038 | -3.751802 | -0.1069543 |
|  |  |  |  |  |  |  |
| Vaccine coverage category |  |  |  |  |  |  |
| <90% | Comparator |  |  |  |  |  |
| 90%+ | -0.9971429 | 0.2628714 | -3.79 | 0 | -1.512361 | -0.4819245 |
| Missing | -0.3060771 | 0.1919717 | -1.59 | 0.111 | -0.6823347 | 0.0701805 |
|  |  |  |  |  |  |  |
| Latitude | 0.0004414 | 0.0102541 | 0.04 | 0.966 | -0.0196562 | 0.020539 |
| Longitude | -0.0361163 | 0.0139607 | -2.59 | 0.01 | -0.0634788 | -0.0087539 |
| Migrant endemic | 0.2588663 | 0.0421123 | 6.15 | 0 | 0.1763276 | 0.341405 |
| Constant | -51.3146 | 33.91589 | -1.51 | 0.13 | -117.7885 | 15.15932 |
| logs | 5.120404 | 0.3609526 | 0 | 0 | 4.41295 | 5.827858 |

1. Class 2

| **Variable** | **Coef.** | **Std. Err.** | **z** | **P>\|z\|** | **95%LI** | **95%UI** |
| --- | --- | --- | --- | --- | --- | --- |
|  |  |  |  |  |  |  |
| Year | -0.0206478 | 0.0049418 | -4.18 | 0 | -0.0303337 | -0.010962 |
|  |  |  |  |  |  |  |
| Population group |  |  |  |  |  |  |
| First time blood donors | Comparator |  |  |  |  |  |
| General population | 1.005988 | 0.0616885 | 16.31 | 0 | 0.8850803 | 1.126895 |
| MSM | 1.489106 | 0.0584838 | 25.46 | 0 | 1.37448 | 1.603732 |
| PWID | 1.875761 | 0.0760282 | 24.67 | 0 | 1.726748 | 2.024773 |
| Pregnant women | 1.087373 | 0.0591699 | 18.38 | 0 | 0.9714021 | 1.203344 |
| Prisoners | 1.781034 | 0.0641395 | 27.77 | 0 | 1.655323 | 1.906746 |
|  |  |  |  |  |  |  |
| Vaccine coverage category |  |  |  |  |  |  |
| <90% | Comparator |  |  |  |  |  |
| 90%+ | 0.0275825 | 0.0527657 | 0.52 | 0.601 | -0.0758364 | 0.1310014 |
| Missing | -0.2164622 | 0.0715279 | -3.03 | 0.002 | -0.3566543 | -0.07627 |
|  |  |  |  |  |  |  |
| Latitude | 0.008846 | 0.0100104 | 0.88 | 0.377 | -0.010774 | 0.0284661 |
| Longitude | -0.0496403 | 0.0136953 | -3.62 | 0 | -0.0764826 | -0.0227979 |
| Migrant endemic | -0.1884574 | 0.0411525 | -4.58 | 0 | -0.2691148 | -0.1077999 |
| Constant | 38.68212 | 9.860031 | 3.92 | 0 | 19.35681 | 58.00743 |
| logs | 6.49764 | 0.1737184 | 0 | 0 | 6.157158 | 6.838122 |

1. Class 3

| **Variable** | **Coef.** | **Std. Err.** | **z** | **P>\|z\|** | **95%LI** | **95%UI** |
| --- | --- | --- | --- | --- | --- | --- |
|  |  |  |  |  |  |  |
| Year | -0.0233006 | 0.0092402 | -2.52 | 0.012 | -0.0414111 | -0.0051901 |
|  |  |  |  |  |  |  |
| Population group |  |  |  |  |  |  |
| First time blood donors | Comparator |  |  |  |  |  |
| General population | 0.6698777 | 0.1133828 | 5.91 | 0 | 0.4476515 | 0.892104 |
| MSM | 1.523366 | 0.1492726 | 10.21 | 0 | 1.230797 | 1.815935 |
| PWID | 1.360546 | 0.1016856 | 13.38 | 0 | 1.161246 | 1.559846 |
| Pregnant women | 0.7252413 | 0.0985693 | 7.36 | 0 | 0.532049 | 0.9184337 |
| Prisoners | 1.353401 | 0.1078259 | 12.55 | 0 | 1.142066 | 1.564736 |
|  |  |  |  |  |  |  |
| Vaccine coverage category |  |  |  |  |  |  |
| <90% | Comparator |  |  |  |  |  |
| 90%+ | 0.3077653 | 0.0952553 | 3.23 | 0.001 | 0.1210684 | 0.4944622 |
| Missing | -0.8335515 | 0.2817466 | -2.96 | 0.003 | -1.385765 | -0.2813383 |
|  |  |  |  |  |  |  |
| Latitude | -0.0271551 | 0.0063558 | -4.27 | 0 | -0.0396122 | -0.0146981 |
| Longitude | 0.0585678 | 0.0052041 | 11.25 | 0 | 0.0483678 | 0.0687677 |
| Migrant endemic | -0.0277334 | 0.010568 | -2.62 | 0.009 | -0.0484463 | -0.0070205 |
| Constant | 44.2736 | 18.59398 | 2.38 | 0.017 | 7.830075 | 80.71712 |
| logs | 4.099355 | 0.1175387 | 0 | 0 | 3.868984 | 4.329727 |

**Supplementary figure 1:** Predicted HBsAg prevalence vs actual study prevalence (on a log scale) for a) class 1; b) class 2; c) class 3

a) class 1

b) class 2

c) class 3

**Supplementary table 4:** Sensitivity analysis (SA) comparing HBsAg prevalence for the general population, pregnant women, and first-time blood donors (FTBD) when including/excluding studies on migrants in the finite mixture model, and comparing with the multiplication method for migrants

| **Country** | **General population** | **General population SA** | **Pregnant women** | **Pregnant women SA** | **FTBD** | **FTBD_SA** |
| --- | --- | --- | --- | --- | --- | --- |
| Austria | 0.34% (0.32%, 0.35%) | 0.61% (0.57%, 0.64%) | 0.37% (0.36%, 0.39%) | 0.61% (0.57%, 0.65%) | 0.09% (0.09%, 0.10%) | 0.14% (0.13%, 0.14%) |
| Belgium | 0.33% (0.32%, 0.33%) | 0.26% (0.26%, 0.27%) | 0.38% (0.38%, 0.38%) | 0.29% (0.29%, 0.29%) | 0.05% (0.05%, 0.05%) | 0.04% (0.04%, 0.04%) |
| Bulgaria | 2.04% (1.92%, 2.16%) | 1.55% (1.45%, 1.65%) | 2.24% (2.16%, 2.33%) | 1.54% (1.44%, 1.65%) | 0.62% (0.60%, 0.64%) | 0.37% (0.35%, 0.39%) |
| Croatia | 0.61% (0.59%, 0.63%) | 1.29% (1.23%, 1.34%) | 0.68% (0.66%, 0.70%) | 1.28% (1.22%, 1.35%) | 0.17% (0.17%, 0.18%) | 0.30% (0.29%, 0.31%) |
| Cyprus | 6.68% (5.76%, 7.60%) | 2.73% (2.55%, 2.91%) | 6.71% (5.90%, 7.53%) | 2.72% (2.53%, 2.91%) | 0.35% (0.31%, 0.39%) | 0.68% (0.64%, 0.72%) |
| Czechia | 0.39% (0.38%, 0.39%) | 1.87% (1.81%, 1.93%) | 0.45% (0.44%, 0.45%) | 1.86% (1.79%, 1.93%) | 0.06% (0.06%, 0.06%) | 0.45% (0.44%, 0.46%) |
| Denmark | 0.25% (0.25%, 0.25%) | 0.85% (0.70%, 0.99%) | 0.29% (0.29%, 0.30%) | 1.16% (0.96%, 1.35%) | 0.04% (0.04%, 0.04%) | 0.05% (0.05%, 0.06%) |
| Estonia | 0.82% (0.78%, 0.86%) | 1.46% (1.37%, 1.55%) | 0.90% (0.87%, 0.94%) | 1.45% (1.35%, 1.55%) | 0.23% (0.23%, 0.24%) | 0.35% (0.33%, 0.36%) |
| Finland | 0.16% (0.12%, 0.20%) | 0.39% (0.33%, 0.45%) | 0.18% (0.14%, 0.21%) | 0.39% (0.32%, 0.45%) | 0.04% (0.04%, 0.05%) | 0.09% (0.07%, 0.10%) |
| France | 0.39% (0.39%, 0.39%) | 0.33% (0.33%, 0.33%) | 0.45% (0.45%, 0.46%) | 0.36% (0.36%, 0.36%) | 0.06% (0.06%, 0.06%) | 0.06% (0.06%, 0.06%) |
| Germany | 0.20% (0.19%, 0.21%) | 0.32% (0.31%, 0.32%) | 0.22% (0.21%, 0.23%) | 0.35% (0.35%, 0.35%) | 0.05% (0.05%, 0.06%) | 0.05% (0.05%, 0.05%) |
| Greece | 2.26% (2.19%, 2.32%) | 2.50% (2.42%, 2.58%) | 2.48% (2.43%, 2.53%) | 2.49% (2.40%, 2.58%) | 0.69% (0.68%, 0.70%) | 0.62% (0.60%, 0.64%) |
| Hungary | 1.38% (1.32%, 1.43%) | 2.22% (2.15%, 2.28%) | 1.52% (1.48%, 1.55%) | 2.21% (2.13%, 2.29%) | 0.41% (0.40%, 0.41%) | 0.54% (0.53%, 0.56%) |
| Iceland | 0.09% (0.05%, 0.12%) | 0.56% (0.42%, 0.71%) | 0.09% (0.06%, 0.11%) | 0.77% (0.58%, 0.97%) | 0.00% (0.00%, 0.00%) | 0.03% (0.03%, 0.04%) |
| Ireland | 0.13% (0.10%, 0.17%) | 0.47% (0.39%, 0.55%) | 0.14% (0.11%, 0.16%) | 0.65% (0.53%, 0.76%) | 0.00% (0.00%, 0.01%) | 0.03% (0.03%, 0.03%) |
| Italy | 0.75% (0.73%, 0.78%) | 1.61% (1.55%, 1.66%) | 0.83% (0.81%, 0.85%) | 1.60% (1.54%, 1.66%) | 0.22% (0.21%, 0.22%) | 0.38% (0.37%, 0.39%) |
| Latvia | 0.89% (0.84%, 0.93%) | 1.53% (1.44%, 1.61%) | 0.98% (0.95%, 1.01%) | 1.52% (1.43%, 1.62%) | 0.26% (0.25%, 0.26%) | 0.36% (0.35%, 0.38%) |
| Liechtenstein | 0.05% (0.04%, 0.06%) | 0.99% (0.68%, 1.29%) | 0.05% (0.04%, 0.06%) | 1.34% (0.92%, 1.77%) | 0.01% (0.01%, 0.01%) | 0.06% (0.05%, 0.08%) |
| Lithuania | 1.41% (1.33%, 1.49%) | 2.25% (2.17%, 2.34%) | 1.55% (1.50%, 1.60%) | 2.24% (2.15%, 2.34%) | 0.42% (0.40%, 0.43%) | 0.55% (0.54%, 0.57%) |
| Luxembourg | 0.25% (0.20%, 0.31%) | 0.49% (0.41%, 0.57%) | 0.26% (0.22%, 0.29%) | 0.68% (0.57%, 0.79%) | 0.01% (0.01%, 0.01%) | 0.03% (0.03%, 0.03%) |
| Malta | 1.45% (1.40%, 1.50%) | 2.22% (2.14%, 2.30%) | 1.60% (1.56%, 1.64%) | 2.21% (2.12%, 2.30%) | 0.43% (0.42%, 0.44%) | 0.54% (0.53%, 0.56%) |
| Netherlands | 0.24% (0.24%, 0.24%) | 0.25% (0.25%, 0.25%) | 0.28% (0.28%, 0.28%) | 0.28% (0.28%, 0.28%) | 0.03% (0.03%, 0.03%) | 0.04% (0.04%, 0.04%) |
| Norway | 0.27% (0.27%, 0.28%) | 0.23% (0.17%, 0.30%) | 0.32% (0.31%, 0.33%) | 0.32% (0.24%, 0.41%) | 0.04% (0.04%, 0.04%) | 0.01% (0.01%, 0.02%) |
| Poland | 0.42% (0.41%, 0.43%) | 2.20% (2.13%, 2.28%) | 0.49% (0.48%, 0.50%) | 2.19% (2.11%, 2.28%) | 0.06% (0.06%, 0.06%) | 0.54% (0.52%, 0.55%) |
| Portugal | 0.57% (0.55%, 0.59%) | 1.46% (1.41%, 1.52%) | 0.63% (0.61%, 0.65%) | 1.46% (1.39%, 1.52%) | 0.16% (0.16%, 0.16%) | 0.35% (0.33%, 0.36%) |
| Romania | 2.87% (2.74%, 3.01%) | 3.17% (3.06%, 3.27%) | 3.15% (3.07%, 3.23%) | 3.16% (3.04%, 3.27%) | 0.89% (0.87%, 0.92%) | 0.80% (0.78%, 0.83%) |
| Slovakia | 0.47% (0.46%, 0.48%) | 2.44% (2.36%, 2.52%) | 0.54% (0.53%, 0.55%) | 2.43% (2.34%, 2.52%) | 0.07% (0.07%, 0.07%) | 0.60% (0.59%, 0.62%) |
| Slovenia | 0.40% (0.38%, 0.42%) | 0.67% (0.64%, 0.71%) | 0.45% (0.43%, 0.46%) | 0.67% (0.63%, 0.71%) | 0.11% (0.11%, 0.11%) | 0.15% (0.15%, 0.16%) |
| Spain | 0.51% (0.50%, 0.51%) | 0.43% (0.42%, 0.43%) | 0.59% (0.58%, 0.59%) | 0.47% (0.47%, 0.47%) | 0.07% (0.07%, 0.07%) | 0.07% (0.07%, 0.07%) |
| Sweden | 0.15% (0.11%, 0.20%) | 0.19% (0.19%, 0.19%) | 0.15% (0.12%, 0.19%) | 0.21% (0.21%, 0.21%) | 0.01% (0.00%, 0.01%) | 0.03% (0.03%, 0.03%) |
| United Kingdom | 0.19% (0.19%, 0.19%) | 0.15% (0.15%, 0.16%) | 0.22% (0.22%, 0.23%) | 0.17% (0.17%, 0.17%) | 0.03% (0.03%, 0.03%) | 0.03% (0.03%, 0.03%) |

**Supplementary table 5:** Sensitivity analysis (SA) comparing HBsAg prevalence for men who have sex with men (MSM), people who inject drugs (PWID), prisoners, and migrants when including/excluding studies on migrants in the finite mixture model, and comparing with the multiplication method for migrants

| **Country** | **MSM** | **MSM SA** | **PWID** | **PWID SA** | **Prisoners** | **Prisoners SA** | **Migrant** | **Migrant SA** |
| --- | --- | --- | --- | --- | --- | --- | --- | --- |
| Austria | 1.59% (1.54%, 1.65%) | 2.17% (2.01%, 2.34%) | 1.20% (1.18%, 1.22%) | 1.51% (1.47%, 1.56%) | 1.18% (1.15%, 1.22%) | 1.79% (1.68%, 1.89%) | 2.15% | 4.70% (4.57%, 4.82%) |
| Belgium | 0.80% (0.79%, 0.80%) | 0.63% (0.63%, 0.64%) | 1.59% (1.58%, 1.61%) | 1.64% (1.63%, 1.65%) | 1.35% (1.34%, 1.36%) | 1.07% (1.06%, 1.07%) | 2.80% | 4.61% (4.60%, 4.62%) |
| Bulgaria | 7.91% (7.62%, 8.21%) | 5.07% (4.59%, 5.54%) | 6.23% (6.12%, 6.34%) | 3.64% (3.50%, 3.78%) | 6.16% (5.96%, 6.36%) | 4.24% (3.95%, 4.52%) | 2.24% | 10.07% (9.72%, 10.42%) |
| Croatia | 2.77% (2.69%, 2.84%) | 4.30% (4.06%, 4.54%) | 2.10% (2.07%, 2.13%) | 3.06% (3.00%, 3.13%) | 2.08% (2.03%, 2.12%) | 3.58% (3.43%, 3.73%) | 0.76% | 8.70% (8.57%, 8.84%) |
| Cyprus | 0.01% (0.00%, 0.01%) | 8.28% (7.74%, 8.83%) | 1.13% (1.06%, 1.20%) | 6.10% (5.88%, 6.31%) | 0.01% (0.00%, 0.02%) | 7.02% (6.62%, 7.43%) | 3.60% | 15.42% (14.98%, 15.86%) |
| Czechia | 0.94% (0.93%, 0.95%) | 5.97% (5.62%, 6.32%) | 1.86% (1.84%, 1.88%) | 4.32% (4.24%, 4.39%) | 1.58% (1.56%, 1.59%) | 5.01% (4.83%, 5.19%) | 2.93% | 11.62% (11.48%, 11.76%) |
| Denmark | 0.62% (0.61%, 0.63%) | 0.33% (0.23%, 0.44%) | 1.25% (1.23%, 1.26%) | 0.01% (0.00%, 0.02%) | 1.05% (1.04%, 1.07%) | 0.20% (0.05%, 0.35%) | 2.89% | 4.03% (3.82%, 4.23%) |
| Estonia | 3.58% (3.49%, 3.68%) | 4.80% (4.50%, 5.11%) | 2.74% (2.69%, 2.79%) | 3.44% (3.34%, 3.54%) | 2.71% (2.63%, 2.79%) | 4.01% (3.80%, 4.22%) | 2.13% | 9.60% (9.31%, 9.89%) |
| Finland | 0.79% (0.66%, 0.91%) | 1.43% (1.21%, 1.65%) | 0.58% (0.50%, 0.67%) | 0.99% (0.86%, 1.12%) | 0.58% (0.48%, 0.68%) | 1.17% (0.98%, 1.36%) | 3.73% | 3.19% (2.85%, 3.54%) |
| France | 0.95% (0.94%, 0.95%) | 0.78% (0.77%, 0.78%) | 1.88% (1.86%, 1.90%) | 2.00% (1.98%, 2.01%) | 1.59% (1.59%, 1.60%) | 1.30% (1.30%, 1.31%) | 3.39% | 5.48% (5.47%, 5.49%) |
| Germany | 0.98% (0.95%, 1.02%) | 0.75% (0.75%, 0.76%) | 0.73% (0.72%, 0.74%) | 1.93% (1.92%, 1.94%) | 0.72% (0.07%, 0.74%) | 1.26% (1.25%, 1.27%) | 2.51% | 5.33% (5.31%, 5.34%) |
| Greece | 8.60% (8.41%, 8.79%) | 7.70% (7.28%, 8.11%) | 6.80% (6.75%, 6.85%) | 5.64% (5.56%, 5.73%) | 6.72% (6.61%, 6.84%) | 6.51% (6.29%, 6.74%) | 4.11% | 14.48% (14.32%, 14.64%) |
| Hungary | 5.65% (5.51%, 5.80%) | 6.94% (6.56%, 7.32%) | 4.39% (4.35%, 4.43%) | 5.06% (4.99%, 5.13%) | 4.34% (4.26%, 4.43%) | 5.85% (5.66%, 6.04%) | 2.97% | 13.24% (13.10%, 13.39%) |
| Iceland | 0.00% (0.00%, 0.00%) | 0.22% (0.13%, 0.31%) | 0.01% (0.01%, 0.01%) | 0.00% (0.00%, 0.01%) | 0.00% (0.00%, 0.00%) | 0.13% (0.02%, 0.25%) | 2.80% | 2.80% (2.45%, 3.15%) |
| Ireland | 0.00% (0.00%, 0.00%) | 0.18% (0.11%, 0.25%) | 0.02% (0.01%, 0.02%) | 0.00% (0.00%, 0.01%) | 0.00% (0.00%, 0.00%) | 0.11% (0.02%, 0.20%) | 1.82% | 2.38% (2.19%, 2.57%) |
| Italy | 3.33% (3.24%, 3.42%) | 5.23% (4.93%, 5.52%) | 2.54% (2.51%, 2.57%) | 3.76% (3.69%, 3.83%) | 2.51% (2.46%, 2.56%) | 4.37% (4.22%, 4.53%) | 4.00% | 10.34% (10.25%, 10.44%) |
| Latvia | 3.85% (3.76%, 3.95%) | 5.00% (4.70%, 5.30%) | 2.95% (2.91%, 3.00%) | 3.59% (3.50%, 3.68%) | 2.92% (2.84%, 3.00%) | 4.18% (3.98%, 4.38%) | 2.53% | 9.95% (9.69%, 10.21%) |
| Liechtenstein | 0.24% (0.20%, 0.28%) | 0.39% (0.21%, 0.57%) | 0.18% (0.15%, 0.20%) | 0.01% (0.00%, 0.02%) | 0.18% (0.15%, 0.20%) | 0.24% (0.02%, 0.46%) | 0.09% | 4.61% (3.89%, 5.32%) |
| Lithuania | 5.76% (5.60%, 5.93%) | 7.03% (6.62%, 7.43%) | 4.48% (4.42%, 4.54%) | 5.13% (5.04%, 5.21%) | 4.43% (4.32%, 4.54%) | 5.93% (5.71%, 6.15%) | 2.54% | 13.39% (13.16%, 13.62%) |
| Luxembourg | 0.00% (0.00%, 0.00%) | 0.19% (0.12%, 0.26%) | 0.03% (0.03%, 0.04%) | 0.00% (0.00%, 0.01%) | 0.00% (0.00%, 0.00%) | 0.12% (0.03%, 0.20%) | 1.86% | 2.48% (2.32%, 2.65%) |
| Malta | 5.92% (5.74%, 6.10%) | 6.94% (6.51%, 7.38%) | 4.61% (4.55%, 4.66%) | 5.06% (4.95%, 5.17%) | 4.55% (4.46%, 4.65%) | 5.85% (5.62%, 6.09%) | 2.40% | 13.25% (13.10%, 13.40%) |
| Netherlands | 0.60% (0.59%, 0.60%) | 0.61% (0.60%, 0.61%) | 1.21% (1.20%, 1.22%) | 1.58% (1.57%, 1.59%) | 1.02% (1.01%, 1.03%) | 1.03% (1.02%, 1.03%) | 2.96% | 4.45% (4.44%, 4.46%) |
| Norway | 0.68% (0.66%, 0.69%) | 0.09% (0.05%, 0.13%) | 1.36% (1.34%, 1.38%) | 0.00% (0.00%, 0.01%) | 1.15% (1.13%, 1.17%) | 0.05% (0.00%, 0.10%) | 3.39% | 1.25% (1.04%, 1.46%) |
| Poland | 1.02% (1.01%, 1.04%) | 6.89% (6.48%, 7.31%) | 2.02% (2.00%, 2.04%) | 5.02% (4.93%, 5.11%) | 1.71% (1.69%, 1.74%) | 5.81% (5.60%, 6.02%) | 1.92% | 13.17% (12.98%, 13.36%) |
| Portugal | 2.58% (2.49%, 2.67%) | 4.81% (4.51%, 5.12%) | 1.96% (1.92%, 1.99%) | 3.45% (3.36%, 3.54%) | 1.93% (1.89%, 1.98%) | 4.02% (3.85%, 4.19%) | 5.00% | 9.62% (9.52%, 9.73%) |
| Romania | 10.50% (10.23%, 10.77%) | 9.41% (8.87%, 9.95%) | 8.38% (8.30%, 8.45%) | 6.98% (6.86%, 7.09%) | 8.29% (8.12%, 8.46%) | 8.01% (7.73%, 8.30%) | 1.31% | 17.18% (16.94%, 17.42%) |
| Slovakia | 1.13% (1.11%, 1.15%) | 7.52% (7.08%, 7.97%) | 2.22% (2.19%, 2.24%) | 5.51% (5.42%, 5.60%) | 1.88% (1.86%, 1.91%) | 6.36% (6.14%, 6.59%) | 1.24% | 14.20% (14.01%, 14.39%) |
| Slovenia | 1.88% (1.82%, 1.94%) | 2.38% (2.20%, 2.57%) | 1.42% (1.39%, 1.44%) | 1.67% (1.62%, 1.71%) | 1.40% (1.36%, 1.44%) | 1.96% (1.85%, 2.07%) | 1.05% | 5.11% (4.98%, 5.24%) |
| Spain | 1.22% (1.21%, 1.23%) | 1.00% (1.00%, 1.01%) | 2.38% (2.36%, 2.41%) | 2.54% (2.52%, 2.55%) | 2.03% (2.02%, 2.04%) | 1.67% (1.66%, 1.68%) | 1.96% | 6.78% (6.77%, 6.79%) |
| Sweden | 0.00% (0.00%, 0.00%) | 0.46% (0.45%, 0.46%) | 0.02% (0.02%, 0.02%) | 1.20% (1.19%, 1.21%) | 0.00% (0.00%, 0.00%) | 0.77% (0.76%, 0.79%) | 3.50% | 3.46% (3.44%, 3.49%) |
| United Kingdom | 0.48% (0.48%, 0.48%) | 0.37% (0.37%, 0.38%) | 0.97% (0.96%, 0.98%) | 0.99% (0.99%, 1.00%) | 0.82% (0.81%, 0.83%) | 0.64% (0.63%, 0.64%) | 3.29% | 2.91% (2.90%, 2.92%) |

**Supplementary table 6:** Comparing how countries are separated into classes when including/excluding studies on migrants in the finite mixture model

| **Country** | **Class** | **Class in sensitivity analysis** |
| --- | --- | --- |
| Cyprus | 1 | 3 |
| Iceland | 1 | 1 |
| Ireland | 1 | 1 |
| Luxembourg | 1 | 1 |
| Sweden | 1 | 2 |
| Belgium | 2 | 2 |
| Czechia | 2 | 3 |
| Denmark | 2 | 1 |
| France | 2 | 2 |
| Netherlands | 2 | 2 |
| Norway | 2 | 1 |
| Poland | 2 | 3 |
| Slovakia | 2 | 3 |
| Spain | 2 | 2 |
| United Kingdom | 2 | 2 |
| Austria | 3 | 3 |
| Bulgaria | 3 | 3 |
| Croatia | 3 | 3 |
| Estonia | 3 | 3 |
| Finland | 3 | 3 |
| Germany | 3 | 2 |
| Greece | 3 | 3 |
| Hungary | 3 | 3 |
| Italy | 3 | 3 |
| Latvia | 3 | 3 |
| Liechtenstein | 3 | 1 |
| Lithuania | 3 | 3 |
| Malta | 3 | 3 |
| Portugal | 3 | 3 |
| Romania | 3 | 3 |
| Slovenia | 3 | 3 |
